# A Rare Case of Primary Pleural Neurofibroma

**DOI:** 10.7759/cureus.17062

**Published:** 2021-08-10

**Authors:** Garima Sharma, Sudhir Saxena, Rajkumar Seenivasagam, Swarnava Tarafdar, Ipsit Ilahi

**Affiliations:** 1 Department of Radiology, All India Institute of Medical Sciences, Rishikesh, Rishikesh, IND; 2 Department of Surgical Oncology, All India Institute of Medical Sciences, Rishikesh, Rishikesh, IND

**Keywords:** primary pleural masses, neurogenic tumors, computed tomography, magnetic resonance imaging, intrathoracic neurofibroma

## Abstract

Metastatic deposits are the most common cause of pleural masses, solitary or multiple. Primary pleural neoplasms are rare entities that are occasionally encountered. Of these, the tumors of neurogenic origin are exceedingly rare with only a few cases reported in the literature. We describe a case of an isolated neurofibroma involving the pleura in a 23-year-old male patient, who underwent CT and MRI scans of the thorax as part of the initial investigations. The final diagnosis was clinched by a CT-guided biopsy of the lesion.

## Introduction

While pleura remains an important site for metastatic deposits for intrathoracic malignancies, primary pleural neoplasms are rarely encountered [1]. Of these, malignant tumors are more common than benign ones, malignant mesothelioma being the most common. Other malignant tumors include lymphoma, malignant fibrous tumors, and sarcoma. Solitary fibrous tumor is the most common benign neoplasm, other entities include lipoma, mesothelial cysts, and calcifying fibrous pseudotumor [2]. 

Neurofibromas are benign nerve sheath tumors that can involve peripheral nerves at any level. They can attain large sizes and pose a diagnostic challenge, especially when present in an unusual location. A search through the literature reveals three case reports of primary pleural neurofibroma without any association with neurofibromatosis.

## Case presentation

**Figure 1:**
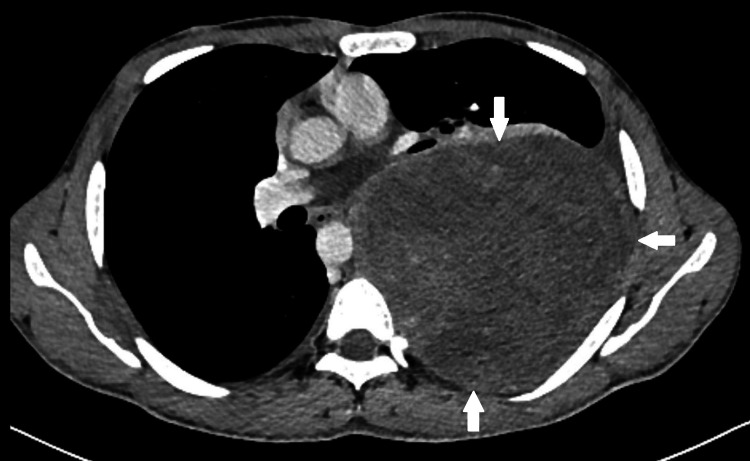
Axial section of contrast-enhanced CT chest shows a large mildly enhancing mass lesion along posterior pleura (white arrows), which is causing mass effect over the left lung parenchyma

A 23-year-old male patient presented to the outpatient department with complaints of left-sided chest pain and difficulty in breathing, which was gradually increasing for one year. There was no significant past medical history. The patient was a non-smoker. System-specific examination revealed reduced air entry on the left side. The rest of the physical examination was unremarkable. For further evaluation, contrast-enhanced chest CT was done, which revealed two closely abutting mildly enhancing soft tissue attenuation mass lesions in the posterior mediastinum on the left side (Figure 1).

**Figure 2:**
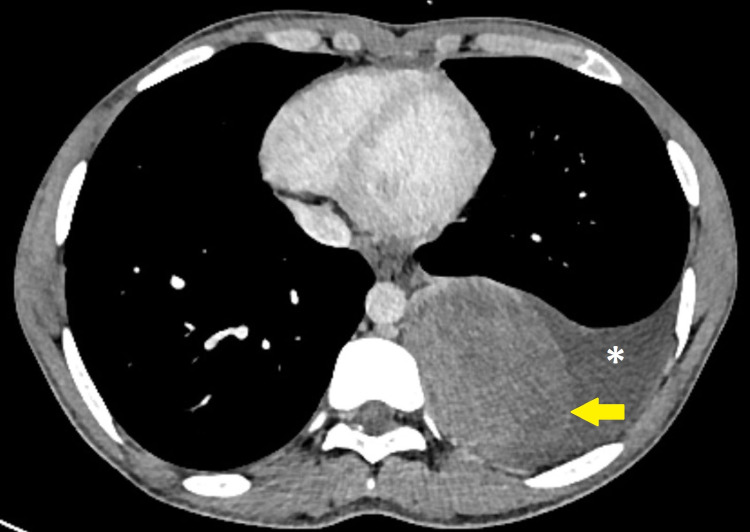
Axial section of contrast-enhanced CT chest shows mass lesion between the two layers of parietal pleura (yellow arrow) and moderate pleural effusion is seen (white asterisk)

The lesions were located between the visceral and parietal pleural layers with associated pleural effusion (Figure 2). The larger lesion was causing mass effect over the left lung parenchyma. However, no parenchymal extension was seen. Posteriorly, the lesions were abutting the chest wall with the preservation of extra-pleural fat. There was no evidence of any rib erosion/remodeling. Medially, the lesions were reaching up to the paraspinal region. However, no obvious spinal extension was seen.

**Figure 3:**
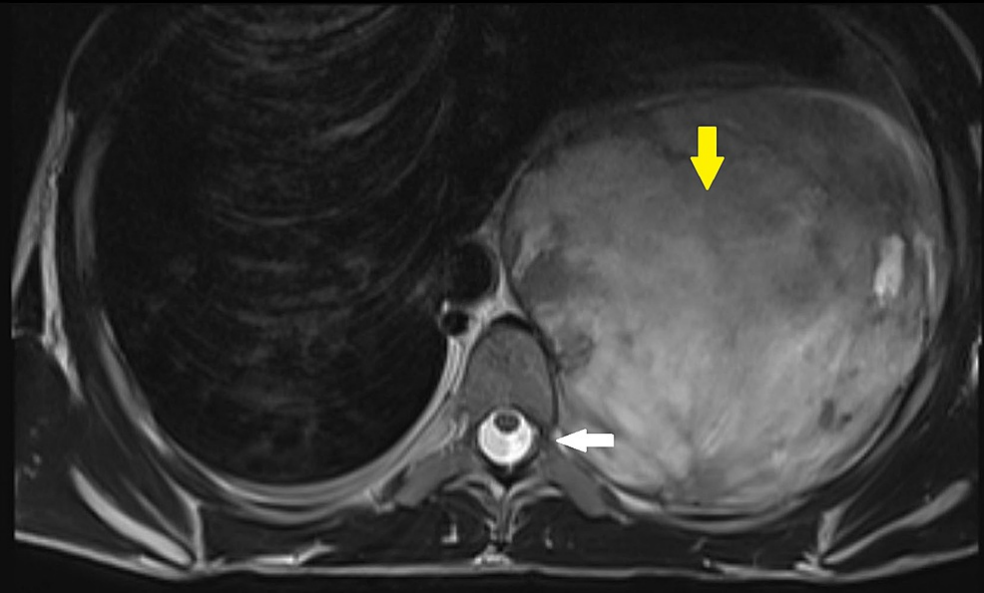
Axial section of T2 weighted MRI shows heterogeneously T2 hyperintense mass lesion (yellow arrow) and no intra-spinal communication was seen (white arrow)

**Figure 4:**
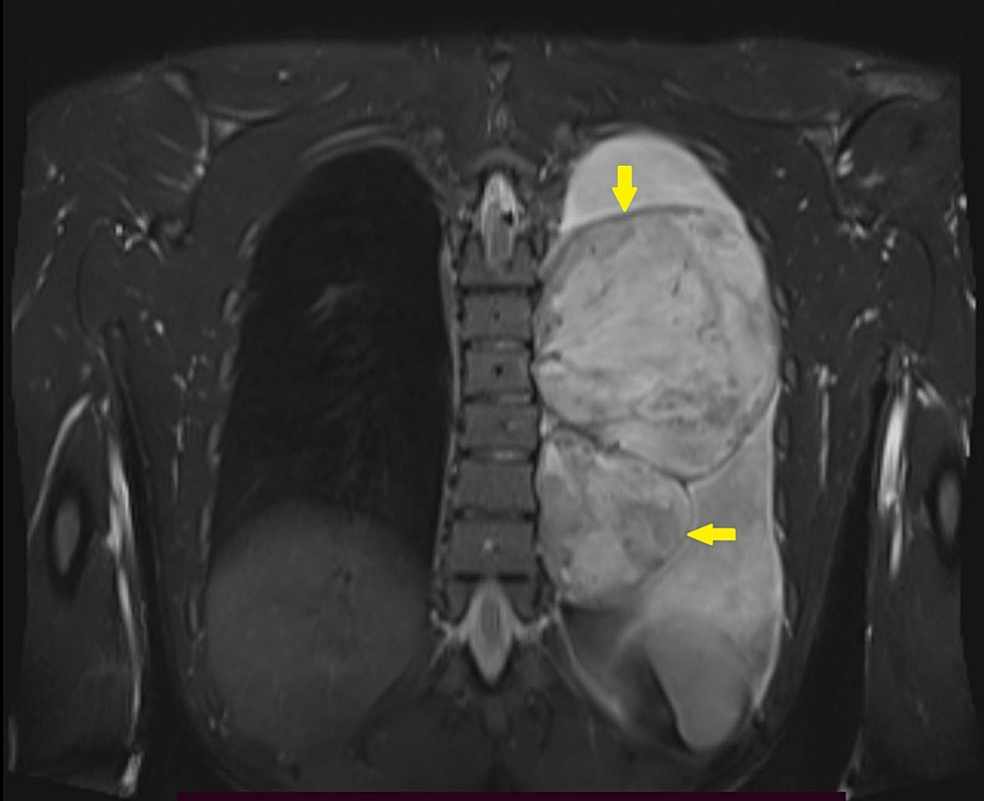
Coronal section of T2 weighted MRI shows two T2 hyperintense pleural-based lesions (yellow arrows) within the left pleural cavity with associated pleural effusion

For confirming the same, the patient underwent MRI. The lesions were hypointense on T1 weighted images and hyperintense on T2 weighted images (Figure 3 and Figure 4). No communication with the spinal canal was seen.

**Figure 5:**
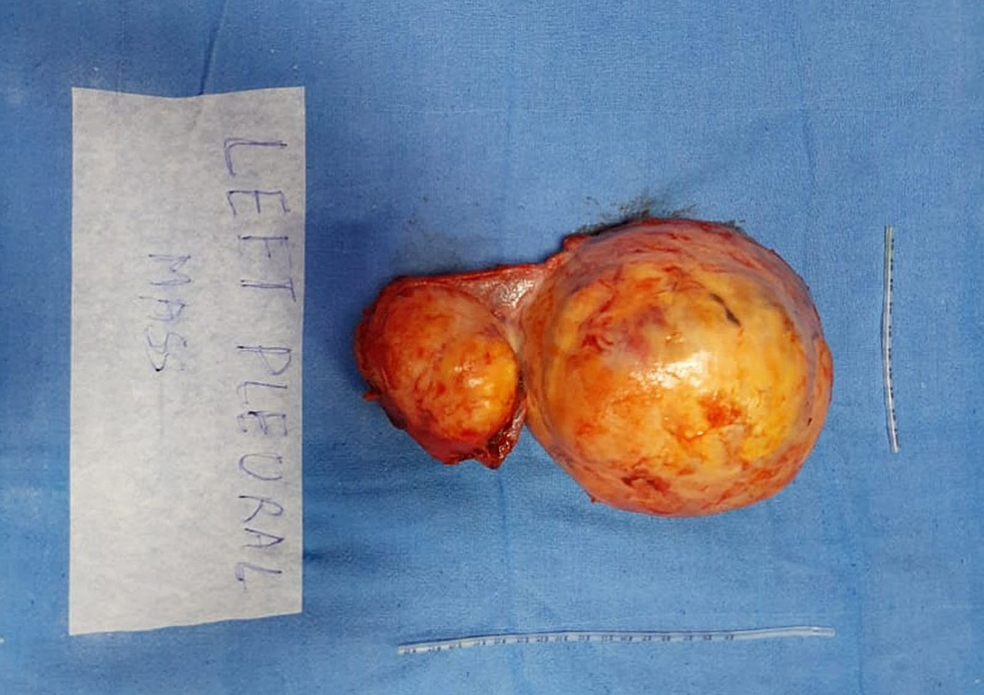
Gross specimen after surgical resection shows well-circumscribed ovoid masses

A CT-guided biopsy was done. Histopathology revealed fascicles of spindle cells and fibrillar eosinophilic cytoplasm. Immunohistochemistry markers study suggested features of neurofibroma. The patient underwent surgical excision of the mass. Intraoperatively, the masses were found to be pleural-based with smooth, encapsulated with well-circumscribed margins (Figure 5). It was well separated from the left lung parenchyma. As suggested by imaging studies, no spinal communication was found intraoperatively. Hence, based on radiological, surgical, and histopathological findings, a definite diagnosis of a primary pleural neurofibroma was made. The patient was discharged after an uneventful postoperative course.

## Discussion

The major differentials for a benign pleural mass include solitary fibrous tumor, benign mesothelioma, mesothelial cysts, lipoma, and calcifying fibrous pseudotumor, with solitary fibrous tumors being the commonest.

Neurofibromas are the most common benign peripheral nerve sheath tumors. They can have localized, diffuse, or plexiform growth patterns. The localized form can occur sporadically. However, diffuse and plexiform forms are associated with neurofibromatosis type I. While mediastinal involvement is common in both adults and children [3], pulmonary and pleural involvement is rarely seen. Due to the presence of peribronchial and periarterial neural plexuses, few cases of intraparenchymal nerve sheath tumors have been reported without any chest wall or pleural connection [4].

The pleura is a serous layer that covers the thoracic structures and is divided into visceral and parietal pleurae. The visceral pleura is innervated by the vagus and sympathetic trunks while the parietal pleura receives its nerve supply from somatic, sympathetic, and parasympathetic fibers, via the intercostals nerves. The diaphragmatic pleura is supplied by the branches of phrenic nerves. Nerve sheath tumors can arise from these nerves with direct pleural involvement. 

Langman et al. [5] reported two cases of primary localized pleural neurofibroma out of which one was a known case of neurofibromatosis type I. Krishnamurthy et al. [6], reported another case of isolated pleural neurofibroma in a 39-year-old female who presented with worsening chest pain. Another case of a 31-year-old male with pleural neurofibroma was reported by Gupta et al. [7].

Schwannoma is another benign peripheral nerve sheath origin tumor with a spindle cell morphology on histopathology. Outside the central nervous system, the flexor surfaces of the extremities or nerve roots are common locations of occurrence. Few cases of pleural schwannomas have been reported [8-9]. 

Presenting symptoms largely depend on the size and location of these tumors and are non-specific (chest pain, dyspnea, and deformities). Those associated with neurofibromatosis will show other systemic manifestations of the syndrome. Pleural effusion mainly occurs due to mass effect and resultant lymphatic obstruction. Cross-sectional imaging plays a crucial role in the diagnosis and management of pleural masses. Careful assessment of the nature of the lesion, origin, and ancillary imaging findings are useful in narrowing the differentials. Localized neurofibromas are best treated with complete surgical resection. To prevent reoccurrence, tumors should be completely excised. 

## Conclusions

Primary pleural neoplasms of neurogenic origin are exceedingly rare. While histopathology is the mainstay for diagnosis, detailed imaging has a significant impact on management. This case report emphasizes the need to be aware of the possibility of such tumors in an uncommon location and of adequate imaging studies before the operative management. Imaging not only delineates the origin but also reveals the relation of the lesion with adjacent structures. Tumors in the paraspinal region of the posterior mediastinum can be easily be confused with a pleural tumor, particularly in a large lesion, where the origin is often obscured. Hence, MRI, in adjunct with CT, is an important modality to assess the intra-spinal extension. 
